# *QuickStats:* Death Rates* from Unintentional Falls Among Adults Aged ≥65 Years, by Sex and Age Group — United States, 2024

**DOI:** 10.15585/mmwr.mm7537a3

**Published:** 2026-09-24

**Figure:**
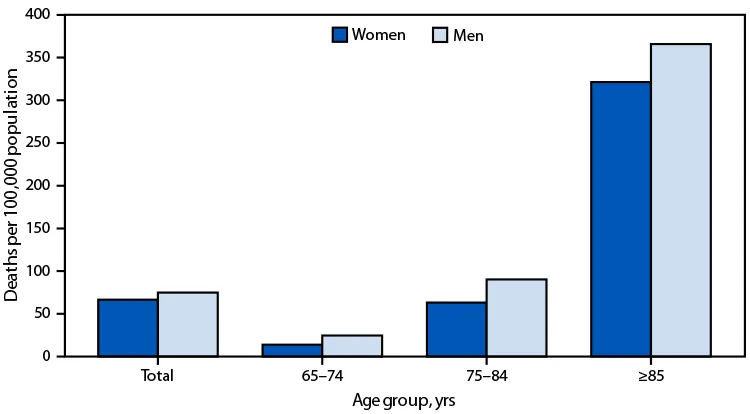
In 2024, the death rates from unintentional falls among adults aged ≥65 years were 74.8 per 100,000 population for men and 66.6 for women. Within each age group, rates were higher for men than for women. Unintentional fall death rates increased with age, with the highest rates among persons aged ≥85 years.

**Supplementary Table:** https://stacks.cdc.gov/view/cdc/259934#tabs-3

**Source: **National Center for Health Statistics, National Vital Statistics System, Mortality Data, 2024. Underlying Cause of Death, 2018–2024, Single Race

